# Applying early divergent characters in higher rank taxonomy of *Melampsorineae* (*Basidiomycota, Pucciniales*)

**DOI:** 10.1080/21501203.2022.2089262

**Published:** 2022-07-11

**Authors:** Peng Zhao, Yan Li, Yuanjie Li, Fang Liu, Junmin Liang, Xin Zhou, Lei Cai

**Affiliations:** a State Key Laboratory of Mycology, Institute of Microbiology, Chinese Academy of Sciences (CAS), Beijing, China; b College of Plant Protection, Jilin Agricultural University, Changchun, China

**Keywords:** Family classification, *Pucciniales*, rust fungi, systematics, taxonomic criteria

## Abstract

Rust fungi in the order *Pucciniales* represent one of the largest groups of phytopathogens, which occur on mosses, ferns to advanced monocots and dicots. Seven suborders and 18 families have been reported so far, however recent phylogenetic studies have revealed para- or polyphyly of several morphologically defined suborders and families, particularly in *Melampsorineae*. In this study, a comprehensive phylogenetic framework was constructed based on a molecular phylogeny inferred from rDNA sequences of 160 species belonging to 16 genera in *Melampsorineae* (i.e. *Chrysomyxa, Cerospora, Coleopuccinia, Coleosporium, Cronartium, Hylospora, Melampsora, Melampsorella, Melampsoridium, Milesina, Naohidemyces, Pucciniastrum, Quasipucciniastrum, Rossmanomyces, Thekopsora, Uredinopsis*). Our phylogenetic inference indicated that 13 genera are monophyletic with strong supports, while *Pucciniastrum* is apparently polyphyletic. A new genus, *Nothopucciniastrum* was therefore established and segregated from *Pucciniastrum*, with ten new combinations proposed. At the family level, this study further demonstrates the importance of applying morphologies of spore-producing structures (basidia, spermogonia, aecia, uredinia and telia) in higher rank taxonomy, while those traditionally applied spore morphologies (basidiospores, spermatia, aeciospores, urediniospores and teliospores) represent later diverged characters that are more suitable for the taxonomy at generic and species levels. Three new families, *Hyalopsoraceae, Nothopucciniastraceae* and *Thekopsoraceae* were proposed based on phylogenetic and morphological distinctions, towards a further revision of *Pucciniales* in line with the phylogenetic relationships.

## Introduction

Plant parasitic rusts, taxonomically as members of the order *Pucciniales*, are one of the most diverse groups of fungal pathogens, with over 7 800 species recognised worldwide (Arthur 1934; Hiratsuka et al. 1992; Zhao et al. 2021; Aime et al. 2006; Webster and Weber 2007). Rusts are obligate parasites with up to five different spore types, including basidiospores, spermatia, aeciospores, urediniospores, teliospores, as well as different lifestyles (micro-, hemi-, demi-, or macrocyclic) that occur on a single (autoecious), or alternate between two unrelated host plants (heteroecious) (Cummins and Hiratsuka 1983). Many species are wreaking havoc on agricultural and forest crop plants, resulting in significant economic losses (Cummins and Hiratsuka 2003). Due to the serious threats posed to these crops, *Puccinia* spp. on wheat and *Melampsora lini* on flax were listed in the “top 10 most important fungal pathogens” in a recent global survey of important plant pathogens (Dean et al. 2012). Furthermore, many rust species, such as *Austropuccinia psidii* (myrtle rust), *Cronartium ribicola* (white pine blister rust), *Hemileia vastatrix* (coffee rust), *Phakopsora pachyrhizi* (Asian soybean rust) etc., are known to be among the most important and threatening species to agriculture and forestry (Ono et al. 1992; Beenken 2014, 2017). Despite the importance of rust fungi, their taxonomy is still debated due to the lack of information on host alternation, and morphological characteristics of their various spore stages (Kuprevich and Tranzschel 1957; Savile 1976; Aime 2006).

The taxonomic ranks of plant-parasitic rusts vary at the generic and suprageneric levels. Rust fungi have been classified into different classes (*Basidiomycetes, Pucciniomycetes* or *Urediniomycetes*) or orders (*Pucciniales* or *Uredinales*) at various time periods (Plowright 1889; Dietel 1928; Cummins 1971; Hiratsuka et al. 1992). Rust fungi were recently found to be monophyletic, and the order *Pucciniales* was proposed to accommodate these plant parasitic rusts (Aime et al. 2006; Hibbett et al. 2007; Zhao et al. 2016). At the family level, the traditional taxonomy of rust fungi changed dramatically over time, and they were divided into 2–14 families based on criteria applied at the time. At early 20^th^ century, all rusts were initially divided into 2–4 families on the basis of the presence of teliospore pedicels and basidial formation (Dietel 1928; Arthur 1934; Hiratsuka 1955). Such a taxonomic treatment has been widely debated, as many apparently unrelated genera have been placed in the same family simply because of morphological similarities in their teliospores (Wilson and Henderson 1966; Leppik 1972; Hiratsuka and Sato 1982). Later, spermogonial morphology was introduced as criterion and the taxonomic importance of this criterion at the family level was further evaluated (Hiratsuka and Cummins 1963; Savile 1976). Hiratsuka and Cummins summarised 12 morphological types of spermogonia in rust fungi and categorised them into six major groups based on the structure of spermogonia (Cummins and Hiratsuka 1983). Thus, a taxonomic scheme with 14 families was proposed and uredinologists universally accepted this taxonomic classification (Hiratsuka and Cummins 1983; Hiratsuka et al. 1992; Cummins and Hirastuka 2003). However, several defined families, such as *Chaconiaceae, Pucciniaceae, Pucciniastraceae, Pucciniosiraceae* and *Uropyxidaceae*, have been revealed to be poly- or paraphyletic in molecular phylogenetic studies (Maier et al. 2003; Wingfield et al. 2004; Aime 2006). Aime (2006) has roughly divided the order *Pucciniales* into three suborders based on molecular phylogeny: *Melampsorineae, Mikronegeriineae* and *Uredinineae*, but the polyphyly of several morphologically defined families remains unresolved (Maier et al. 2003; Wingfield et al. 2004; Beenken et al. 2012; Beenken and Wood 2015; Qi et al. 2019). Thereafter, Aime and McTaggart (2021) presented a high-rank classification of the *Pucciniales*, in which the order was divided into seven suborders and 18 families. Among these families, all rusts with sessile teliospores were classified in the suborder *Melampsorineae*, with 16 genera included in four families, *Coleosporiaceae, Melampsoraceae, Milesinaceae* and *Pucciniastraceae* based on their aecial similarities. However, these families showed significant morphological differences in the structures of spermogonia and telia, which have long been employed as the main criteria for family classification (Cummins and Hiratsuka 1983, 2003). As a result, the taxonomic placement of these 16 genera remains a point of contention, and it is necessary to reassign those genera in the proper family in the suborder *Melampsorineae*.

In this study, we conducted molecular phylogenetic analyses and morphological reappraisal of *Melampsorineae*. The objectives of this study were: (1) to evaluate the monophyly of traditional morphologically defined genera and determine their familial placements and generic boundaries in *Melampsorineae*; (2) to propose a taxonomic amendment towards establishing monophyletic families in *Melampsorineae*.

## Materials and methods

### Molecular phylogeny and supergeneric-level delimitation

We have included sequence data from our previous taxonomic studies on genera in *Melampsorineae* (Zhao et al. 2014, 2015, 2016, 2017, 2020, 2021; Qi et al. 2019) as well as some newly generated sequence data from our one unpublished paper (under review), and detailed information of specimens, host species and GenBank accession numbers has been listed in Table 1. In addition, rDNA sequence data from previous taxonomic studies on *Pucciniales*, particularly those in *Melampsorineae*, were included in the final alignment (Table 1). Those sequences were acquired from taxonomic references and retrieved from NCBI (https://www.ncbi.nlm.nih.gov/) based on the accession number. To determine the phylogenetic relationships of genera in the suborder *Melampsorineae*, rDNA ITS and LSU sequences of representative taxa from the genera *Chrysomyxa, Coleopuccinia, Coleosporium, Cronartium, Hylospora, Melampsora, Melampsorella, Melampsoridium, Milesina, Naohidemyces, Pucciniastrum, Quasipucciniastrum, Rossmanomyces, Thekopsora*, and *Uredinopsis* were chosen for phylogenetic studies (Table 1). Two *Gymnosporangium* species were selected as outgroups. The majority of the sequence data came from samples that had detailed morphological information.

**Table 1.** rDNA sequence data from selected genera in the *Melampsorineae* suborder of *Pucciniales* order used for phylogenetic studies.

Family	Genus	Species	Specimen No.^a^	Spore stage	Host	Country	GenBank Accession No.^a^		Reference
							ITS	LSU	
***Cronartiaceae***	***Cronartium***	*Cronartium appalachianum*	Ca-1	0, I	*Pinus virginiana*	USA	L76484	—	Vogler & Bruns (1998)
		*Cronartium arizonicum*	MICH253346	II, III	*Castilleja linariaefolia*	USA	MK208284	MK193824	**Qi et al. (2019)**
			MICH301493	0, I	*Pinus ponderosa*	USA	OM746343	OM746511	Present study
			FSprP- 1	0, I	*Pinus ponderosa*	USA	L76504	—	Vogler & Bruns (1998)
		*Cronartium armandii*	ZP-R901	II, III	*Ribes* sp.	China	OM746349	OM746517	Present study
			HMAS45350	0, I	*Pinus armandii*	China	MZ520620	MZ520623	**Present study**
		*Cronartium bethelii*	MICH253453	II, III	*Quercus emoryi*	USA	OM746353	OM746521	**Present study**
			FLAS-F-16608	0, I	*Pinus palustris*	USA	OM746355	OM746523	**Present study**
			CrKor-1	0, I	*Pinus strobus*	USA	L76497	—	Vogler & Bruns (1998)
		*Cronartium coleosporioides*	CsSr-1	0, I	*Pinus contorta*	USA	L76500	—	Vogler & Bruns (1998)
			SHmC-9	0, I	*Pinus contorta*	USA	L76511	—	Vogler & Bruns (1998)
			SPC-21	0, I	*Pinus contorta var. latifolia*	USA	L76513	—	Vogler & Bruns (1998)
		*Cronartium comandrae*	HMAS24619	II, III	*Comandra richardsiana*	Canada	OM746358	OM746526	**Present study**
			ISC392261	II, III	*Comandra pallida*	USA	OM746362	OM746530	**Present study**
			MICH253330	II, III	*Comandra pallida*	USA	OM746365	OM746533	**Present study**
			MICH253516	II, III	*Comandra pallida*	USA	OM746367	OM746535	**Present study**
			MICH253517	II, III	*Comandra pallida*	USA	OM746368	OM746536	**Present study**
			UBC-F5867	II, III	*Comandra pallida*	USA	OM746370	OM746538	**Present study**
			BCpC-15	0, I	*Pinus contorta*	USA	L76477	—	Vogler & Bruns (1998)
			CPeEl-1	0, I	*Pinus eldarica*	USA	L76481	—	Vogler & Bruns (1998)
			NYBG36363	0, I	*Pinus* sp.	USA	OM746374	OM746542	**Present study**
			NYBG36381	0, I	*Pinus* sp.	USA	OM746375	OM746543	**Present study**
		*Cronartium comptoniae*	UBC-F5871	II, III	*Comptonia peregrina*	USA	OM746377	OM746545	**Present study**
			UBC-F5870	II, III	*Comptonia asplenitolia*	USA	OM746378	OM746546	**Present study**
			MICH253506	0, I	*Pinus banksiana*	Canada	OM746382	OM746550	**Present study**
		*Cronartium flaccidum*	FLAS-F-55559	II, III	*Paeonia officinalis*	Finland	OM746383	OM746551	**Present study**
			HMAS37551	II, III	*Paeonia lactiflora*	China	OM746389	OM746557	**Present study**
			HMAS89231	II, III	*Paeonia lactiflora*	China	MK208289	MK193822	**Present study**
			HMAS44164	0, I	*Pinus taiwanensis*	China	MK208288	MK193816	**Present study**
		*Cronartium floridanum*	MICH299992	0, I	*Pinus palustris*	USA	OM746408	OM746576	**Present study**
			MICH300092	0, I	*Pinus palustris*	USA	OM746409	OM746577	**Present study**
		*Cronartium fusiforme*	HMAS56356	II, III	*Quercus variabilis*	China	OM746415	OM746583	**Present study**
			HMAS71281	II, III	*Quercus variabilis*	China	OM746416	OM746584	**Present study**
			HMAS9043	II, III	*Quercus emoryii*	USA	OM746418	OM746586	**Present study**
			HMAS35526	II, III	*Quercus* sp.	China	OM746419	OM746587	**Present study**
			HMAS52087	0, I	*Pinus silvestris*	China	OM746420	OM746588	**Present study**
		*Cronartium keteleeriae*	HMAS638	0, I	*Keteleeria davidiana*	China	OM746422	—	**Present study**
			HMAS11129	0, I	*Keteleeria davidiana*	China	OM746421	OM746588	**Present study**
			HMAS638	0, I	*Keteleeria davidiana*	China	OM746422	—	**Present study**
		*Cronartium occidentale*	MICH253479	II, III	*Ribes gandfalii*	USA	OM746429	OM746594	**Present study**
			MICH253477	II, III	*Ribes odoratum*	USA	OM746430	OM746595	**Present study**
			MICH253481	II, III	*Ribes aureum*	USA	OM746431	OM746596	**Present study**
		*Cronartium orientale*	HMAS242642	II, III	*Quercus aquifolioides*	China	OM746433	OM746599	**Present study**
			HMAS77666	II, III	*Quercus liaotungensis*	China	OM746435	OM746601	**Present study**
			HMAS77667	II, III	*Quercus liaotungensis*	China	OM746436	OM746602	**Present study**
			HMAS82717	II, III	*Quercus glandulifera*	China	MK208292	MK193817	**Qi et al. (2019)**
		*Cronartium pini*	Cclone 3	II, III	*Melampyrum* sp.	Finland	JF713709	—	Kaitera et al. (2011)
			Crust 1	0, I	*Pinus sylvestris*	Finland	KJ959593	—	Kaitera et al. (2015)
			Crust 2	0, I	*Pinus sylvestris*	Finland	KJ959594	—	Kaitera et al. (2015)
		*Cronartium pyriforme*	MICH253420	0, I	*Pinus contorta*	USA	OM746451	OM746617	**Present study**
			MICH253360	II, III	*Comandra pallida*	USA	OM746452	OM746618	**Present study**
		*Cronartium quercuum*	MICH253529	II, III	*Quercus rubra*	Canada	OM746455	OM746621	**Present study**
			ISC395258	II, III	*Quercus imbricaria*	USA	OM746461	OM746627	**Present study**
			CqE9WM-FP	0, I	*Pinus banksiana*	USA	JN943197	—	Schoch et al. (2012)
			40CR-PNB-LP1	0, I	*Pinus* sp.	Canada	JN943249	—	Schoch et al. (2012)
		*Cronartium ribicola*	UBC-F5886	II, III	*Ribes bracteosum*	USA	OM746479	OM746645	**Present study**
			TSH-17009	II, III	*Ribes sativum*	Japan	OM746480	OM746646	**Present study**
			NYBG267056	II, III	*Ribes* sp.	Canada	OM746483	OM746649	**Present study**
			MICH253525	II, III	*Ribes nigrum*	Romania	OM746484	OM746650	**Present study**
			TSH-1094	II, III	*Pinus coronata*	Japan	OM746491	OM746657	**Present study**
			UBC-F5879	0, I	*Pinus monticola*	Canada	OM746492	OM746658	**Present study**
		*Cronartium strobilinum*	807-ISFSL-FP	0, I	*Pinus* sp.	USA	JN943191	—	Schoch et al. (2012)
			G-317-HGS1-FP	0, I	*Pinus* sp.	USA	JN943192	—	Schoch et al. (2012)
		*Cronartium* sp.1	HMAS41544	II, III	*Saussurea bullockii*	China	OM746501	OM746667	**Present study**
		*Cronartium* sp.2	HMAS40888	II, III	*Ribes aureum*	Germany	—	OM746665	**Present study**
			HMAS49226	II, III	*Ribes aureum*	USA	OM746500	OM746666	**Present study**
		*Cronartium* sp.3	CFB22250	0, I	*Pinus banksiana*	USA	DQ206982	AY700193	Matheny et al. (2006)
			PUR N11655	II, III	*Quercus muehlenbergii*	USA	KY587788	—	Abbasi et al. (2017)
			CqL-1	II, III	*Lithocarpus densiflorus*	USA	L76489	—	Vogler & Bruns (1998)
			CqQ-1	II, III	*Quercus agrifolia*	USA	L76490	—	Vogler & Bruns (1998)
		*Cronartium* sp.4	MICH253424	II, III	*Quercus rubra*	Canada	OM746457	OM746623	**Present study**
		*Cronartium* sp.5	HMAS244165	0, I	*Pinus taiwanensis*	China	OM746503	—	**Present study**
		*Cronartium* sp.6	HMAS18841	II, III	*Castanea* sp.	China	OM746356	OM746524	**Present study**
			HMAS8970	0, I	*Pinus ponderosa*	USA	OM746357	OM746525	**Present study**
		*Cronartium* sp.7	HMAS242639	II, III	*Quercus mongolica*	China	OM746423	—	**Present study**
			ZP-R7	II, III	*Quercus mongolica*	China	OM746424	OM746589	**Present study**
		*Cronartium* sp.8	MICH301494	II, III	*Pinus murrayana*	USA	OM746425	OM746590	**Present study**
		*Cronartium* sp.9	MICH253485	II, III	*Myrica asplenifolia*	Canada	OM746427	OM746592	**Present study**
			MICH253505	II, III	*Myrica gale*	Canada	OM746428	OM746593	**Present study**
		*Cronartium* sp.10	NYBG267052	II, III	*Pinus* sp.	USA	OM746443	OM746609	**Present study**
			NYBG267053	II, III	*Pinus strobus*	Canada	MK208298	MK193829	**Qi et al. (2019)**
			NYBG267051	II, III	*Ribes nigrum*	USA	MK208296	MK193828	**Qi et al. (2019)**
		*Cronartium* sp.11	HMAS56423	II, III	*Quercus aliena*	China	OM746453	OM746619	**Present study**
			HMAS74356	II, III	*Quercus aliena*	China	OM746454	OM746620	**Present study**
		*Cronartium* sp.12	HMAS52871	II, III	*Ribes nigrum*	China	OM746496	OM746662	**Present study**
			HMAS172046	II, III	*Ribes nigrum*	China	OM746497	OM746663	**Present study**
			FLAS-F-16581	0, I	*Pinus taeda*	USA	OM746498	OM746664	**Present study**
Family *incertae sedis*	*Quasipucciniastrum*	*Quasipucciniastrum agrimoniae*	HMAS67301	II, III	*Agrimonia pilosa*	China	MK208261	MK193832	**Qi et al. (2019)**
			HMAS63888	II, III	*Agrimonia pilosa*	China	MK208266	MK193837	**Qi et al. (2019)**
			HMAS13479	II, III	*Agrimonia pilosa*	China	MK208268	MK193839	**Qi et al. (2019)**
			HMAS67309	II, III	*Agrimonia pilosa*	China	MK208263	MK193834	**Qi et al. (2019)**
			HMAS67306	II, III	*Agrimonia pilosa*	China	MK208262	MK193833	**Qi et al. (2019)**
			HMAS24481	II, III	*Agrimonia pilosa*	China	MK208267	MK193838	**Qi et al. (2019)**
			HMAS172173	II, III	*Agrimonia pilosa*	China	MK208265	MK193836	**Qi et al. (2019)**
			HMAS248095	II, III	*Agrimonia pilosa*	China	MK208281	MK193852	**Qi et al. (2019)**
			HMAS172175	II, III	*Agrimonia pilosa*	China	MK208272	MK193843	**Qi et al. (2019)**
			HMAS77430	II, III	*Agrimonia pilosa*	China	MK208271	MK193842	**Qi et al. (2019)**
			HMAS67302	II, III	*Agrimonia pilosa*	China	MK208273	MK193844	**Qi et al. (2019)**
			HMAS82312	II, III	*Agrimonia pilosa*	China	MK208264	MK193835	**Qi et al. (2019)**
*Chrysomyxaceae*	*Chrysomyxa*	*Chrysomyxa arctostaphyli*	CFB22246	II, III	―	―	DQ200930	AY700192	AFTOL-ID 442
		*Chrysomyxa cassandrae*	QFB 25019	II, III	*Chamaedaphne calyculata*	Canada	GU049446	GU049529	Feau et al. (2011)
			813CHC-PC-LP4	0, I	*Picea mariana*	Canada	GU049450	GU049531	Feau et al. (2011)
		*Chrysomyxa chiogenis*	QFB 25026	II, III	*Gaultheria hispidula*	Canada	GU049452	GU049532	Feau et al. (2011)
			631CHS-GAH-ZM28	II, III	*Gaultheria hispidula*	Canada	GU049453	GU049533	Feau et al. (2011)
		*Chrysomyxa empetri*	QFB 25015	II, III	*Empetrum nigrum*	Canada	GU049434	GU049526	Feau et al. (2011)
		*Chrysomyxa ledi*	4D10	0, I	*Picea abies*	Finland	HM037711	HM037707	Kaitera et al. (2010)
			240709	0, I	*Picea abies*	Finland	HM037708	HM037703	Kaitera et al. (2010)
		*Chrysomyxa ledicola*	195CHO_PCM_X1c	0, I	*Picea mariana*	Canada	GU049417	GU049520	Feau et al. (2011)
			24CHO_LEG_RW1	0, I	*Picea mariana*	Canada	GU049418	FJ666446	Feau et al. (2011)
		*Chrysomyxa nagodhii*	QFB 25006	II, III	*Rhododendron groenlandicum*	Canada	GU049431	GU049524	Feau et al. (2011)
			201CH_LE_LE2	II, III	*Ledum glandulosumc*	Canada	GU049427	J666450	Feau et al. (2011)
		*Chrysomyxa neoglandulosi*	DAOM 229530	II, III	*Ledum glandulosumc*	Canada	GU049498	GU049550	Feau et al. (2011)
		*Chrysomyxa piperiana*	DAFVP 14997	II, III	*Ledum macrophyllumc*	Canada	GU049497	GU049565	Feau et al. (2011)
		*Chrysomyxa purpurea*	BJFC-R02299	0, I	*Picea purpurea*	Sichuan, China	NR_158401	MW063518	Cao et al. (2017)
			BJFC-R02300	II, III	*Rhododendron oreodoxa*	China	KX225402	MW898418	Cao et al. (2017)
			BJFC-R01698	II, III	*Rhododendron oreodoxa*	China	KX225401	—	Cao et al. (2017)
			BJFC-R02448	II, III	*Rhododendron oreodoxa*	China	MK770362	MK874622	Cao et al. (2017)
			BJFC-R01699	II, III	*Rhododendron oreodoxa*	China	KX225405	—	Cao et al. (2017)
			HMAS55188	0, I	*Picea purpurea*	China	KX225403	MW898421	Yang (2015)
			BJFC-R02623	II, III	*Rhododendron* sp.	China	MK770364	—	Yang (2015)
			BJFC-R02302	0, I	*Picea purpurea*	China	MK770363	MK874623	Yang (2015)
		*Chrysomyxa rhododendri*	DAFVP14606	II, III	*Ledum lapponicumc*	Canada	GU049467	GU049560	Feau et al. (2011)
		*Chrysomyxa vaccinii*	DAOM 45774	II, III	*Vaccinium parvifolium*	Canada	GU049463	GU049561	Feau et al. (2011)
			DAVFP 10115	II, III	*Vaccinium parvifolium*	Canada	GU049465	GU049562	Feau et al. (2011)
		*Chrysomyxa woroninii*	QFB 25025	II, III	*Ledum groenlandicum*	Canada	GU049462	GU049540	Feau et al. (2011)
		*Chrysomyxa zhuoniensis*	BJFC-R02733	II, III	*Rhododendron* sp.	China	MK770374	MK874636	Yang (2015)
	*Rossmanomyces*	*Rossmanomyces monesis*	DAOM 221982	II, III	*Pyrola uniflora*	Canada	GU049476	GU049547	Feau et al. (2011)
			DAVFP 14528	0, I	*Picea sitchensis*	Canada	GU049479	GU049566	Feau et al. (2011)
		*Rossmanomyces pyrolae*	Only DNA extraction	II, III	*Pyrola asarifolia*	Canada	GU049481	GU049558	Feau et al. (2011)
			DAOM 214476	II, III	*Pyrola minor*	Canada	GU049483	GU049553	Feau et al. (2011)
			QFB 16517	0, I	*Picea* sp.	Canada	GU049484	GU049554	Feau et al. (2011)
			Herbarium 12886	0, I	*Picea glauca*	Canada	GU049485	GU049555	Feau et al. (2011)
			Herbarium CPP	0, I	*Picea glauca*	Canada	GU049486	GU049556	Feau et al. (2011)
			QFB 25055	0, I	*Picea glauca*	Canada	GU049487	—	Feau et al. (2011)
			QFB 25057	II, III	*Pyrola* sp.	Canada	GU049489	—	Feau et al. (2011)
			390CHP-PCG-VF1	II, III	*Pyrola* sp.	Canada	—	FJ666456	Vialle et al. (2013)
*Coleosporiaceae*	*Coleosporium*	*Coleosporium asterum*	BPI 879270	II, III	*Solidago* sp.	USA	GU058009	—	Dixon (unpublished)
			RS1325	II, III	*Solidago* sp.	Canada	HQ317530	—	Beenken et al. (2017)
			N43	II, III	*Kalimeris* sp.	Japan	KX386012	KX386044	**Zhao (unpublished)**
		*Coleosporium cacaliae*	LB09281 / ZT-Myc-58004	II, III	*Adenostyles aliariae*	Switzerland	KY810462	—	Beenken et al. (2017)
			LB09265 / ZT-Myc-58002	II, III	*Campanula latifolia*	Switzerland	KY810467	—	Beenken et al. (2017)
		*Coleosporium clematidis*	N81	II, III	*Clematis* sp.	Japan	KX386007	KX386039	**Zhao (unpublished)**
			N99	II, III	*Clematis* sp.	Japan	KX386008	KX386040	**Zhao (unpublished)**
			N79	II, III	*Clematis* sp.	Japan	KX386010	KX386042	**Zhao (unpublished)**
		*Coleosporium delicatulum*	BPI 877848	II, III	*Solidago* sp.	USA	MF769637	—	McTaggart et al. (2018)
			BPI 871731	II, III	*Symphyotrichum novae-angliae*	USA	MF769638	—	McTaggart et al. (2018)
		*Coleosporium inulae*	GBOL / KR-M-0024937	0, I	*Pinus sylvestris*	Germany	KY783673	—	Beenken et al. (2017)
			LB09168 / ZT-Myc-57996	II, III	*Inula salicina*	Switzerland	KY810470	—	Beenken et al. (2017)
		*Coleosporium montanum*	BPI 877858	II, III	*Solidago* sp.	USA	MF769635	—	McTaggart et al. (2018)
			BPI 877849	II, III	*Solidago* sp.	USA	MF769636	—	McTaggart et al. (2018)
			WU:43601	II, III	*Symphyotrichum novae-angliae*	Austria	MW284589	—	Voglmayr (2020)
		*Coleosporium petasitidis*	LB09254 / ZT-Myc-58000	II, III	*Petasites hybridus*	Switzerland	KY810471	—	Beenken et al. (2017)
		*Coleosporium phellodendri*	N7	II, III	*Phellodendron amurense*	Japan	KX386015	KX386047	**Zhao (unpublished)**
			TSH-R8823	II, III	*Solidago* sp.	Russia	KX386016	KX386048	Zhao et al. (2017)
		*Coleosporium plectranthi*	N16	II, III	*Phellodendron amurense*	Japan	KX386009	KX386041	**Zhao (unpublished)**
			N85	II, III	*Phellodendron amurense*	Japan	KX386011	KX386043	**Zhao (unpublished)**
		*Coleosporium plumeriae*	GDPR 2-4	II, III	*Plumeria* sp.	Caribbean	KF879087	—	Beenken et al. (2017)
			BPI 880744	II, III	*Plumeria* sp.	—	KY764063	—	Demers et al. (unpublished)
			BPI 844177	II, III	*Plumeria* sp.	Guyana	MF769645	—	McTaggart (2018)
			U50	II, III	*Plumeria rubra*	Guyana	MG907225	—	Vialle (2013)
		*Coleosporium senecionis*	PDD-98309	II, III	*Senecio* sp.	New Zealand	KJ716348	—	Beenken et al. (2017)
		*Coleosporium solidaginis*	BPI 863448	II, III	*Solidago* sp.	USA	DQ354559	—	Aime (2006)
			BPI 877855	II, III	*Solidago* sp.	USA	MF769649	—	McTaggart et al. (2018)
			BPI 877852	II, III	*Solidago* sp.	USA	MF769652	—	McTaggart et al. (2018)
		*Coleosporium tussilaginis*	PDD 93250	II, III	*Brachyglottis huntii*	New Zealand	KX985766	—	McTaggart et al. (2018)
			BPI 843412	II, III	*Senecio triangularis*	USA	MF769653	—	McTaggart et al. (2018)
			MCA2389	II, III	*Sonchus* sp.	USA	MG907228	—	Vialle et al. (2018)
		*Coleosporium vernoniae*	BPI 877867	II, III	*Elephantopus tomentosus*	USA	MF769654	—	McTaggart et al.(2017)
			BPI 877869	II, III	*Vernonia* sp.	USA	MF769655	—	McTaggart et al. (2017)
		*Coleosporium zanthoxyli*	KUS-F29608	II, III	*Zanthoxylum planispinum*	South Korea	MH465095	MH460677	Kim (unpublished)
			KUS-F25423	II, III	*Zanthoxylum planispinum*	South Korea	MH465096	MH460678	Kim (unpublished)
*Thekopsoraceae*	*Thekopsora*	*Thekopsora areolata*	LR 4B	0, I	*Picea abies*	Norway	DQ445892	—	Hietala et al. (2008)
			SPL3 5	0, I	*Picea abies*	Norway	DQ445893	—	Fossdal et al. (unpublished)
			Spl 3 5	0, I	*Picea abies*	Norway	DQ087229	—	Hietala et al. (2008)
			Thek 2	0, I	*Picea abies*	Norway	DQ087230	—	Hietala et al. (2008)
			Ru 4	0, I	*Prunus padus*	Norway	DQ087231	—	Hietala et al. (2008)
			2_0910	0, I	*Picea engelmannii*	Finland	KJ546897	KJ546894	Kaitera et al. (2010)
*Melampsoraceae*	*Ceropsora*	*Ceropsora weirii*	574CHW_X_MA7	0, I	*Picea* sp.	Canada	GU049473	GU49544	Feau et al. (2011)
			912CHW_PCG_BU3	0, I	*Picea* sp.	Canada	GU049474	GU49545	Feau et al. (2011)
	*Melampsora*	*Melampsora abietis-canadensis*	666X-TSC-SH11	II, III	—	Canada	EU808020	FJ666512	Feau et al. (2009)
			1399MEA-POG-USA	II, III	*Populus grandidentata*	USA	JN881733	JN934918	Vialle et al. (2013)
		*Melampsora aecidioides*	—	II, III	*Populus alba*	Canada	EU808021	FJ666510	Feau et al. (2009)
		*Melampsora albertensis*	BPI 0021209	II, III	—	USA	JX416848	JX416843	Vialle et al. (2013)
		*Melampsora allii-populina*	1260MEAP-POC-HU	II, III	*Populus canadensis*	—	JN881728	JN934902	Vialle et al. (2013)
		*Melampsora apocyni*	LYR3	II, III	*Apocynum venetum*	China	KR296802	KR296803	Gao et al. (unpublished)
		*Melampsora arctica*	HMAS 8629	II, III	*Salix iliensis*	China	KX386083	KX386112	Zhao et al. (2017)
		*Melampsora capraearum*	NYS-F-003819	II, III	*Salix caprea*	Germany	KU550034	KU550033	Zhao et al. (2016)
		*Melampsora coleosporioides*	HNMAP3114	II, III	*Salix reinii*	Japan	KF780755	KF780638	Zhao et al. (2015)
		*Melampsora epiphylla*	TSH-R12280	II, III	*Salix sachalinensis*	Japan	KF780787	KF780670	Zhao et al. (2017)
		*Melampsora epitea*	TNS-F-121034	II, III	*Salix viminalis*	Germany	KX386070	KX386097	Zhao et al. (2017)
		*Melampsora euphorbiae*	BPI 871135	II, III	*Euphorbia heterophylla*	—	DQ911599	AF426195	Deadman et al. (unpublished)
		*Melampsora euphorbiae-gerardianae*	BRIP 39560	II, III	*Euphorbia peplus*	—	EF192199	—	Aime et al. (unpublished)
		*Melampsora ferrinii*	PUR N6742	II, III	*Salix babylonica*	USA	KJ136570	KJ136563	Toome (2015)
			SAG-21943/2016	II, III	*Salix* sp.	Chile	KY053852	KY053853	Zapata (2016)
		*Melampsora humilis*	TSH-R7650	II, III	*Salix koriyanagi*	Japan	KF780812	KF780695	Zhao et al. (2017)
		*Melampsora iranica*	HMAAC4055	II, III	*Salix* sp.	China	MK372158	MK372191	Wang et al. (2020)
		*Melampsora kamikotica*	HNMAP3186	II, III	*Chosenia arbutifolia*	China	KF780760	KF780643	Zhao et al. (2015)
		*Melampsora laricis-miyabeana*	TSH-R18314	II, III	*Salix reinii*	Japan	KX386071	—	Zhao et al. (2017)
		*Melampsora laricis-pentandrae*	HNMAP3201	II, III	*Salix pentandra*	China	KF780801	KF780684	Zhao et al. (2015c)
		*Melampsora larici-tremulae*	PFH-99-1	II, III	*Populus tremula*	—	JN881744	JN934956	Vialle et al. (2013)
		*Melampsora magnusiana*	1426MEG-CJ-DSD	II, III	*Chelidonium majus*	Sachsen	GQ479845	JN934927	Vialle et al. (2013)
		*Melampsora medusae f. sp. deltoidis*	98D10	II, III	*Populus euramericana*	South Africa	GQ479307	JN934962	Vialle et al. (2013)
		*Melampsora medusae f. sp. tremuloides*	1028ME-LAL-LJ	II, III	*Larix laricina*	Canada	GQ479883	—	Vialle et al. (2013)
		*Melampsora microsora*	HH-53150	II, III	*Salix subfragilis*	Japan	KF780834	KF780717	Zhao et al. (2015c)
		*Melampsora microspora*	97MP10a	II, III	*Populus nigra*	Iraq	JN881737	JN934931	Vialle et al. (2013)
		*Melampsora nujiangensis*	AAH00-1	II, III	*Pinus alba*	England	AY444772	AY444786	Pei et al. (2005)
		*Melampsora occidentalis*	1366MEPR-POPRURT	II, III	*Populus diversifolia*	China	GQ479899	JN934938	Vialle et al. (2013)
		*Melampsora rostrupii*	O8ZK4	II, III	*Mercurialis annua*	Italy	GQ479320	JN934941	Vialle et al. (2013)
		*Melampsora pakistanica*	BA13c	II, III	*Euphorbia helioscopia*	Pakistan	KX237555	KX237556	Ali et al. (2016)
		*Melampsora pinitorqua*	1367MPI-PNI-FI	0, I	*Pinus sylvestris*	Finland	GQ479897	JN934973	Vialle et al. (2013)
		*Melampsora populnea*	PDD 98363	II, III	*Ricinus communis*	New Zealand	KJ716352		Padamsee and McKenzie (2014)
		*Melampsora pruinosae*	1366MEPR-POPR-UR	II, III	*Populus pruinosa*	Canada	GQ479898	JN934939	Feau et al. (2011)
		*Melampsora pulcherrima*	O8ZK2	II, III	*Mercurialis annua*	Canada	JN934940	GQ479321	Feau et al. (2011)
		*Melampsora ribesii-purpureae*	HMAS62584	II, III	*Salix purpurea*	China	KF780766	KF780649	Zhao et al. (2017)
		*Melampsora ribesii-viminalis*	HNMAP1968	II, III	*Salix viminalis*	China	KX386069	KX386096	Zhao et al. (2017)
		*Melampsora ricini*	PDD 98363	II, III	*Ricinus communis*	—	KJ716352	—	Padamsee (2014)
		*Melampsora rostrupii*	PFH08-3	II, III	*Populus alba*	France	JN881752	JN934981	Vialle et al. (2013)
		*Melampsora salicis-albae*	NWC-06210	II, III	*Salix alba*	England	KF780757	KF780640	Zhao et al. (2015c)
		*Melampsora salicis-argyraceae*	HMAS52984	II, III	*Salix argyracea*	China	KF780733	KF780616	Zhao et al. (2015c)
		*Melampsora salicis-bakko*	HNMAP1710	II, III	*Salix sinica*	China	KC631839	KC685596	Zhao et al. (2015c)
		*Melampsora salicis-cavaleriei*	HMAAC4043	II, III	*Salix serrulatifolia*	China	MK277296	MK277301	Wang et al. (2020)
		*Melampsora salicis-futurae*	TSH-R9620	II, III	*Salix futura*	Japan	KC631860	KC685617	Zhao et al. (2017)
		*Melampsora salicis-purpureae*	HMAS62584	II, III	*Salix purpurea*	China	KF780766	KF780649	Zhao et al. (2017)
		*Melampsora salicis-sinicae*	HNMAP1710	II, III	*Salix sinica*	China	KC631839	KC685596	Zhao et al. (2014)
			HNMAP1716	II, III	*Salix sinica*	China	KC631844	KC685601	Zhao et al. (2015c)
		*Melampsora salicis-triandrae*	HNMAP3181	II, III	*Salix triandra*	China	KF780829	KF780712	Zhao et al. (2017)
		*Melampsora salicis-viminalis*	HMAS38658	II, III	*Salix viminalis*	China	KF780732	KF780615	Zhao et al. (2015c)
		*Melampsora x columbiana*	sn-35	II, III	*Populus angustifolia*	USA	JQ042235		Busby et al. (2012)
		*Melampsora yezoensis*	TSH-R1504	II, III	*Salix jessoensis*	Nagano	KF780806	KF780731	Zhao et al. (2015)
			TSH-R1507	II, III	*Salix jessoensis*	Nagano	KF780832	KF780715	Zhao et al. (2015)
*Pucciniastraceae*	*Melampsorella*	*Melampsorella caryophyllacearum*	PUR 82	II, III	*Cerastium* sp.	USA	—	MG907233	Aime et al. (2018)
			WM 1092	0, I	*Abies alba*	—	—	AF426232	Maier et al. (2003)
	*Pucciniastrum*	*Pucciniastrum circaeae*	TSH-R10187	II, III	*Circaea erubescens*	Japan	AB221456	AB221387	Liang et al. (2006)
			B 2098	II, III	*Circaea lutetiana*	Canada	—	AY74569	Vialle (unpublished)
		*Pucciniastrum epilobii*	MK697276	II, III	*Oenothera acaulis*	—	MK697276	—	Hietala et al. (2008)
			Ru11	II, III	*Epilobium watsonii*	Norway	DQ445907	—	Vialle et al. (2013)
			Ru6	II, III	*Epilobium angustifolium*	USA	DQ445906	—	Vialle et al. (2013)
			PUR N11088	II, III	*Chamaenerion angustifolium*	—	—	MW049277	Aime & McTaggart (2021)
		*Pucciniastrum guttatum*	PDD 91889	II, III	*Galium odoratum*	New Zealand	KJ716345		Padamsee (2014)
		*Pucciniastrum lanpingensis*	BJFC-R00355	0, I	*Pinus sylvestris*	France	KF551225	KF551208	Yang et al. (2015)
		*Pucciniastrum minimum*	LD 1081	II, III	*Vaccinium corymbosum*	Mexico	—	HM439777	Rebollar-Alviter et al. (2011)
			PREM 60245	II, III	*Vaccinium corymbosum*	South Africa	—	GU355675	Yang et al. (2015)
		*Pucciniastrum myosotidii*	PDD 93251	II, III	*Myosotidium hortensia*	New Zealand	—	KJ716347	Padamsee (2014)
		*Pucciniastrum nipponicum*	HMBF-GS-53.1	II, III	*Galium davuricum*	China	KC415792	KC416001	Yang et al. (2015)
			HMBF-GS-54.1	II, III	*Galium aparine*	China	KC415793	KC416003	Yang et al. (2015)
		*Pucciniastrum pustulatum*	PDD 101572	II, III	*Epilobium chlorifolium*	New Zealand	KJ698631	—	Padamsee (2014)
		*Pucciniastrum rubiae*	HMBF-XZ-1.1	II, III	*Rubia cordifolia*	China	KC415802	KC416009	Yang (2015)
		*Pucciniastrum verruculosum*	KUS-F29482	II, III	*Aster tataricus*	Korea	MZ725013	MZ724685	Lee et al. (2021)
*Nothopucciniastraceae*	*Nothopucciniastrum*	*Nothopucciniastrum actinidiae*	TSH-R23801	II, III	*Actinidia arguta*	Japan	AB221446	AB221403	Liang et al. (2006)
		*Nothopucciniastrum boehmeriae*	TSH-R21289	II, III	*Boehmeria tricuspis*	Japan	AB221450	AB221393	Liang et al. (2006)
		*Nothopucciniastrum corni*	TSH-R4273 (IBA7671)	II, III	*Clethra kuosa*	Japan	AB221436	AB221408	Liang et al. (2006)
		*Nothopucciniastrum fagi*	TSH-R21254	II, III	*Fagus crenata*	Japan	AB221424	AB221375	Liang et al. (2006)
			TSH-R10724	II, III	*Fagus crenata*	Japan	AB221425	AB221378	Liang et al. (2006)
		*Nothopucciniastrum hikosanense*	TSH-R4287 (IBA2565)	II, III	*Acer rufinerva*	Japan	AB221441	AB221388	Liang et al. (2006)
			TSH-R4289 (IBA8441)	II, III	*Actinidia rufinerva*	Japan	AB221440	AB221389	Liang et al. (2006)
		*Nothopucciniastrum kusanoi*	HH98635	II, III	*Clethra barbinervis*	Japan	AB221429	AB221400	Liang et al. (2006)
			TSH-R21252	II, III	*Clethra barbinervis*	Japan	AB221430	AB221401	Liang et al. (2006)
		*Nothopucciniastrum miyabeanum*	TSH-R4281 (IBA8721)	II, III	*Viburnum furcatum*	Japan	AB221442	AB221394	Liang et al. (2006)
			TSH-R10202	II, III	*Viburnum furcatum*	Japan	AB221443	AB221397	Liang et al. (2006)
		*Nothopucciniastrum styracinum*	TSH-R t015	II, III	*Styrax japonica*	Japan	AB221431	AB221416	Liang et al. (2006)
		*Nothopucciniastrum tiliae*	TSH-R4294 (IBA7670)	II, III	*Tilia japonica*	Japan	AB221453	AB221414	Liang et al. (2006)
			TSH-R4295 (IBA7878)	II, III	*Tilia japonica*	Japan	AB221454	AB221415	Liang et al. (2006)
		*Nothopucciniastrum yoshinagai*	TSH-R4272 (IBA8430)	II, III	*Stewartia monadelpha*	Japan	AB221434	AB221411	Liang et al. (2006)
			TSH-R4270 (IBA8404)	II, III	*Stewartia monadelpha*	Japan	AB221435	AB221410	Liang et al. (2006)
*Hyalopsoraceae*	*Coleopuccinia*	*Coleopuccinia sinensis*	BJFC-R02506	II, III	*Cotoneaster microphyllus*	China	MF802288	MF802285	Cao et al. (2018)
			BJFC-R02364	II, III	*Cotoneaster rubens*	China	MF802287	MF802284	Cao et al. (2018)
			BJFC-R02506	II, III	*Cotoneaster rubens*	China	MF802286	MF802283	Cao et al. (2018)
	*Hyalopsora*	*Hyalopsora nodispora*	PR # 40	II, III	*Adiantum capillus-veneris*	Pakistan	—	MW899330	Riaz et al. (unpublished)
			BPI 893262	II, III	*Adiantum capillus-veneris*	USA	—	KY798373	Demers (unpublished)
			BPI 893261	II, III	*Adiantum capillus-veneris*	USA	KY798372		Demers (unpublished)
		*Hyalopsora polypodii*	BPI 893256	II, III	*Athyrium attenuatum*	USA	—	KY798367	Demers (unpublished)
			K187058	II, III	—	United Kingdom	MZ159483		Gaya et al. (unpublished)
			PDD 71999	II, III	*Deparia petersenii*	New Zealand	—	KJ698627	Padamsee & McKenzie (2014)
		*Hyalopsora neocheilanthis*	BJFC-R00590	II, III	—	China	MK795975	MK795969	Liang et al. (unpublished)
		*Hyalopsora japonica*	BJFC-R00401	II, III	—	China	MK795974	MK795968	Liang et al. (unpublished)
		*Hyalopsora aspidiotus*	PUR N4641	II, III	—	China	—	MW049264	Liang et al. (unpublished)
		*Hyalopsora* sp. 1	BJFC-R02435	II, III	—	China	MK795976	MK795970	Liang et al. (unpublished)
	*Melampsoridium*	*Melampsoridium alni*	H7019539	II, III	*Alnus mandshurica*	Finland	KF031557	KF031534	McKenzie et al. (2013)
		*Melampsoridium betulinum*	ZP-R490	II, III	*Betula* sp.	China	MK518946	MK518638	Zhao et al. (2021)
			H 6035417	II, III	*Betula pubescens*	Finland	KF031556	KF031539	McKenzie et al. (2013)
			PDD 102645	II, III	*Alnus cordata*	New Zealand	KF031559	KF031544	McKenzie et al. (2013)
		*Melampsoridium hiratsukanum*	PDD 77191	II, III	*Alnus pubescens*	Austria	KF031564	KF031546	McKenzie et al. (2013)
			PDD 78493	II, III	*Alnus incana*	Austria	KF031565	KF031547	McKenzie et al. (2013)
*Milesinaceae*	*Uredinopsis*	*Uredinopsis osmundae*	U856	II, III	*Osmunda* sp.	USA	—	MG907245	Aime et al. (2018)
			U1188	II, III	*Athyrium* sp.	USA	—	MG907244	Aime et al. (2018)
		*Uredinopsis pteridis*	U856	II, III	*Osmunda* sp.	USA	—	KM249869	Aime et al. (2018)
		*Uredinopsis filicina*	U1188	II, III	*Athyrium* sp.	USA	—	MG907244	Aime et al. (2018)
			BRIP 60091	II, III	*Pteridium esculentum*	Australia	—	KM249869	McTaggart et al. (2014)
	*Milesina*	*Milesina blechni*	KR-M-0038519	II, III	*Struthiopteris spicant*	Germany	MH908412	MK302189	Bubner et al. (2019)
		*Milesina carpatica*	KR-M-0043192	II, III	*Dryopteris filix-mas*	Germany	MH908454	—	Bubner et al. (2019)
		*Milesina exigua*	KR-M-0050247	II, III	*Polystichum braunii*	Austria	MH908478	MK302211	Bubner et al. (2019)
		*Milesina feurichii*	KR-M-0043159	II, III	*Asplenium septentrionale*	Germany	MH908476	—	Bubner et al. (2019)
		*Milesina kriegeriana*	KR-M-0048480	II, III	*Dryopteris dilatata*	Germany	MH908452	MK302207	Bubner et al. (2019)
		*Milesina leviuscula*	BRIP 58421	II, III	*Nephrolepis* sp.	Viet Nam	MW049269	KM249868	McTaggart et al. (2014)
		*Milesina murariae*	KR-M-0048133	II, III	*Asplenium ruta-muraria*	Germany	MH908422	MK302194	Bubner et al. (2019)
		*Milesina philippinensis*	BRIP 58421	II, III	*Nephrolepis* sp.	Viet Nam	MW049269	KM249868	McTaggart et al. (2014)
		*Milesina polypodii*	KR-M-0043190	II, III	*Polypodium vulgare*	Germany	MH908415	MK302190	Bubner et al. (2019)
		*Milesina scolopendrii*	KR-M-0049051	II, III	*Asplenium scolopendrium*	Germany	MH908467	MK302209	Bubner et al. (2019)
		*Milesina vogesiaca*	KR-M-0043187	II, III	*Polystichum aculeatum*	Germany	MH908440	MK302202	Bubner et al. (2019)
		*Milesina whitei*	KR-M-0050248	II, III	*Polystichum aculeatum*	Germany	MH908479	MK302212	Bubner et al. (2019)
		*Milesina woodwardiana*	KR-M-0049033	II, III	*Woodwardia radicans*	Spain	MH908474	—	Bubner et al. (2019)
	*Naohidemyces*	*Naohidemyces vaccinii*	WM 1098	II, III	*Vaccinium uliginosum*	—	—	AF426238	Maier et al. (2003)
			MIN 928279	II, III	*Vaccinum* sp.	USA	—	KJ698628	Padamsee (2014)
			BPI 871754	II, III	*Vaccinium ovatum*	Washington, USA	—	DQ354563	Padamsee (2014)

For phylogenetic analyses, raw sequence data were aligned by BioEdit v. 7.0.9 (Hall 1999), and multiple alignments were performed with MAFFT v. 7.394 (Katoh et al. 2017). Ambiguous alignment positions were manually adjusted before the final analyses. Topologies were constructed based on maximum likelihood (ML) analyses using RAxML v. 0.95 (Stamatakis 2006). Bayesian Markov Chain Monte Carlo (MCMC) analyses were performed using MrBayes v. 3.1.2 (Huelsenbeck and Ronquist 2001), and Bayesian posterior probabilities (Bpp) were calculated. In ML and Bayesian analyses, the best-fit substitution model was estimated using Modeltest v. 3.7 (Posada and Crandall 1998).

### Morphological comparison

The type specimens, original descriptions, and other published descriptions of the species involved were compared with detailed morphological traits of species, genus and family from previous literature (e.g. Sydow and Sydow 1915; Kuprevich and Tranzschel 1957; Arthur 1933; Wilson and Henderson 1966; Hiratsuka et al. 1992; Cummins and Hiratsuka 2003; Liang et al. 2006 Liang 2006; Yang 2015). Different spore stages of rust fungi were designated by following Roman numerals according to Cummins and Hiratsuka (1983, 2003): spermagonia/spermatia (0), aecia/aeciospores (I), uredinia/urediniospores (II), telia/teliospores (III), and basidia/basidiospore (IV). We applied the definitions of spore stage and morphological types in the whole life cycle based on Cummins and Hiratsuka (2003).

## Results

For ML and Bayesian analyses, a dataset containing selected species from 16 genera in *Melampsorineae* was used. Phylogenetic trees using the combined dataset yielded higher confident values for the generic level than that of the single locus tree, with 570 bp nucleotide positions for ITS and 800 bp for LSU. An overview of the inferred topology is given in Figure 1. *Melampsorineae* is divided into 16 well-supported clades. The monophylies of several genera in *Melampsorineae*, i.e. *Ceropsora, Chrysomyxa, Coleopuccinia, Cronartium, Coleosporium, Hylospora, Melampsorella, Melampsoridium, Naohidemyces, Quasipucciniastrum*, and *Thekopsora*, were confirmed, in agreement with previous studies (Aime et al. 2018; Qi et al. 2019; Zhao et al. 2020; Aime and McTaggart 2021). *Pucciniastrum*, the type genus of the *Pucciniastraceae*, was split into two different clades. The type species of *Pucciniastrum, P. epilobii* clustered with other nine species (i.e. *P. circaeae, P. guttatum, P. lanpingensis, P. minimum, P. myosotidii, P. nipponicum, P. pustulatum, P. rubiae*, and *P. verruculosum*) in one clade, whilst 10 other *Pucciniastrum* species (i.e. *P. actinidiae, P. boehmeriae, P. corni, P. fagi, P. hikosanense, P. kusanoi, P. miyabeanum, P. styracinum, P. tiliae* and *P. yoshinagai*) were in another distinct clade. Species of *Milesina* and *Uredinopsis* were found in a same clade, with the later merged into the *Milesina* species, forming a sister clade to *M. vogesiaca*.

**Figure 1.**
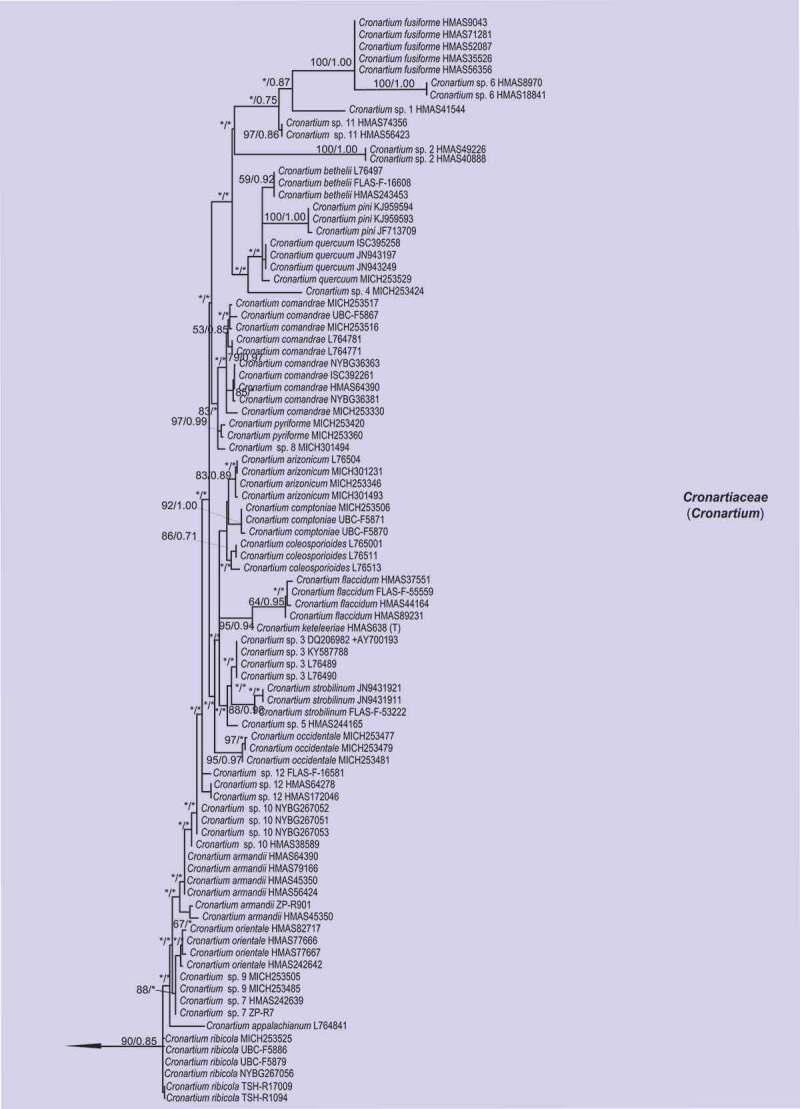
Multilocus phylogenetic tree of the *Melampsorineae* suborder in the *Pucciniales* order. Support values indicated at nodes. Bayesian posterior probabilities ≤ 50% and Maximum Likelihood bootstrap (ML) ≤ 50% were indicated by dash line (–). Family and generic names are listed after each taxon.

**Figure 1.**
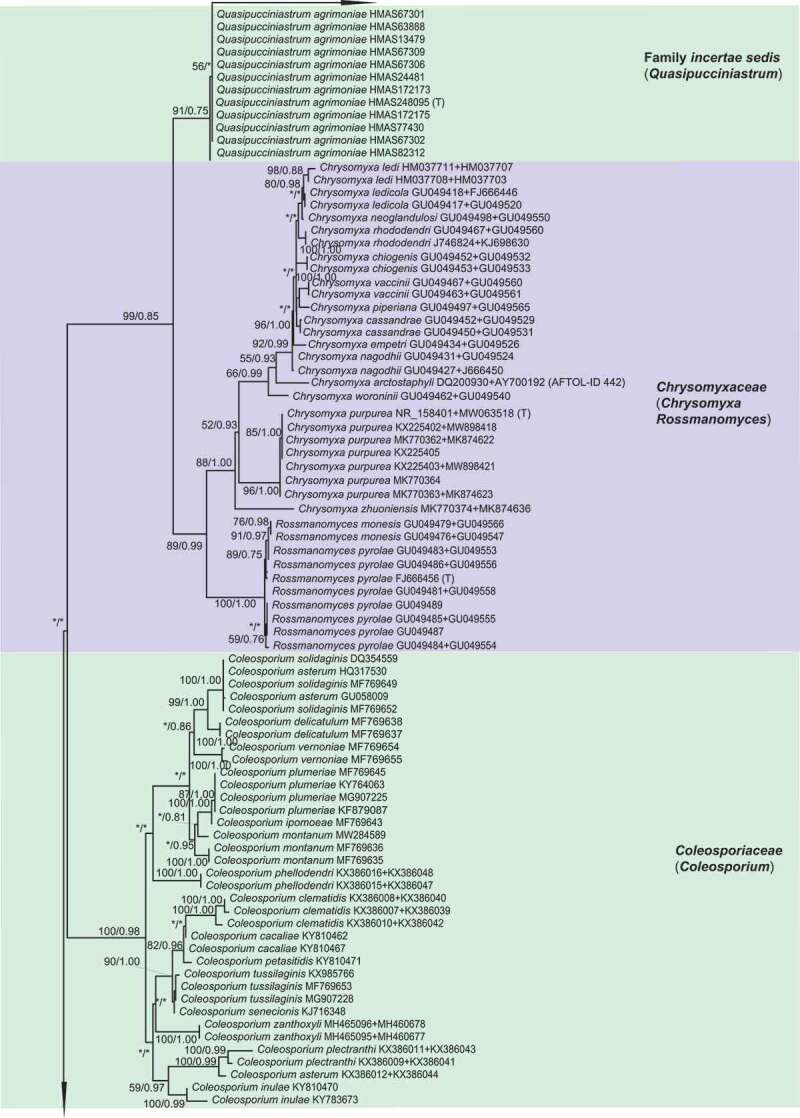
(Continued).

**Figure 1.**
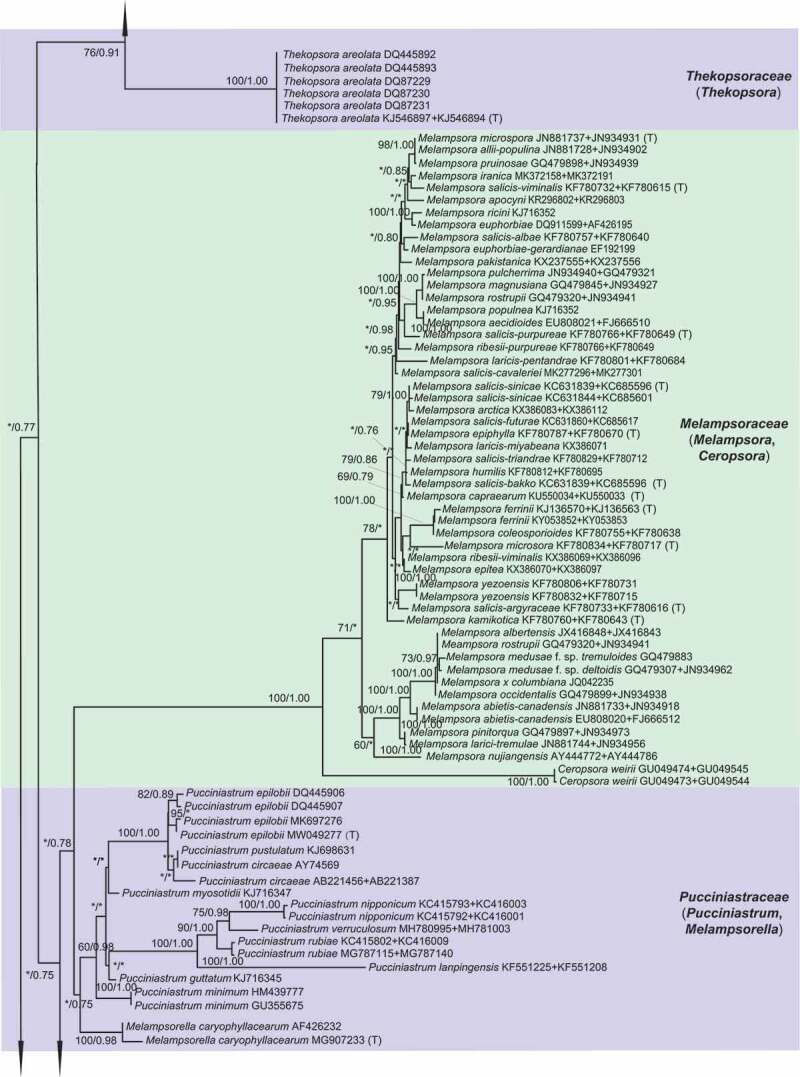
(Continued).

**Figure 1..**
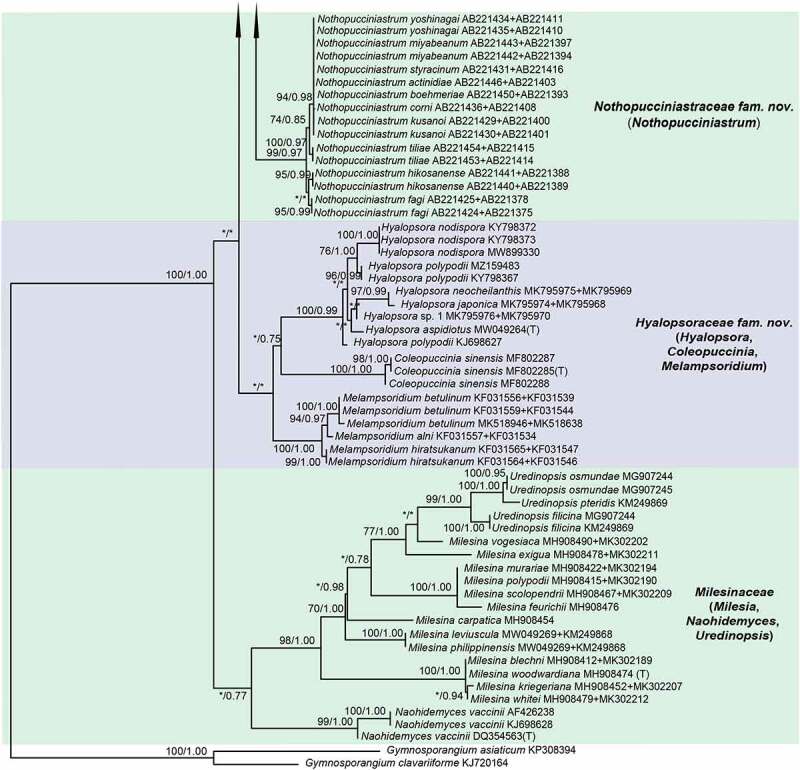
(Continued).

**Figure 2.**
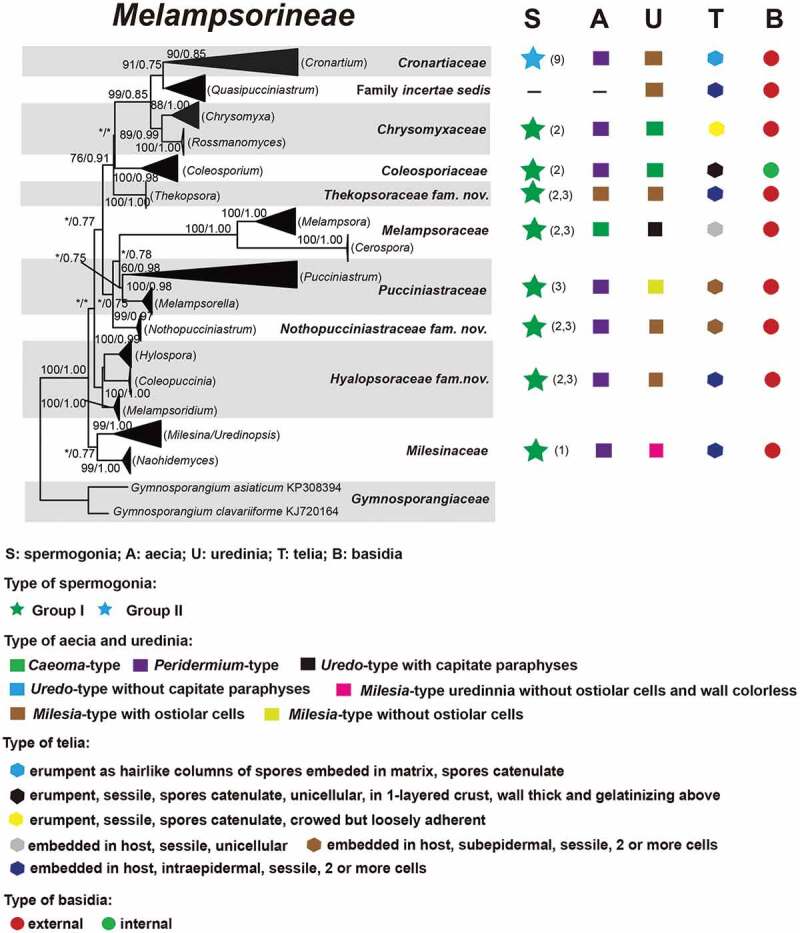
Correlation of phylogeny and morphological criteria used of family delimitation. Dash line (–) in the phylogenetic tree indicated the family or genera lacks of one certain spore stage.

**Figure 3.**
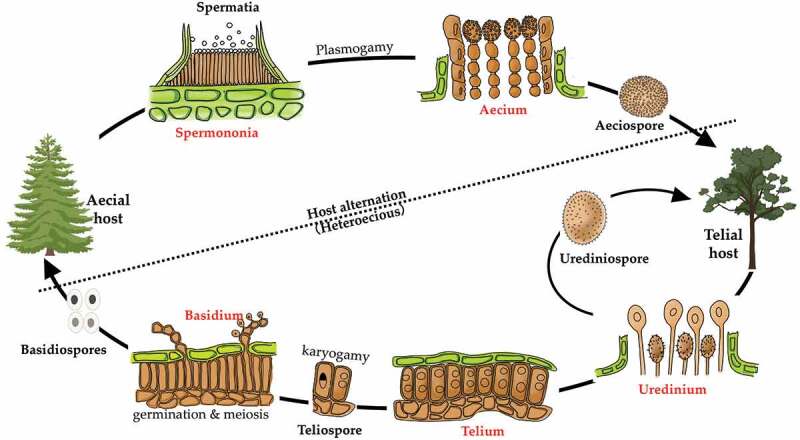
Taxonomic importance of morphological characters in different spore stages during the whole life cycle in the rust fungi is illustrated. The spore-producing structures, i.e. basidia, spermogonia, aecia, uredinia and telia are indicated in red colour and bold, while the spore morphologies, i.e. basidiospores, spermatia, aeciospores, urediniospores and teliospores, are indicated in black colour and bold.

Morphological comparison of each clade is shown in Figure 1. Morphologies in spore-producing structures (i.e. spermogonia, aecia, uredinia, telia and basidia), exhibited the strongest association with the phylogeny. Based on our morphological comparisons and phylogenetic analyses of 16 genera in *Melampsorineae*, ten families, including three new families, *Hyalopsoraceae, Nothopucciniastraceae, Thekopsoraceae*, and one new genus, *Nothopucciniastrum*, were proposed. These families differ from their phylogenetically allied families in their spore-producing structures (basidia, spermogonia, aecia, uredinia and telia), as shown in Figure 2. Previous studies have demonstrated that spore-producing structures were phylogenetically informative at the family level (Zhao et al. 2020, 2021; Aime and McTaggart 2021), and we have further confirmed that these characters throughout the life cycle are of great importance to facilitate natural familial delimitation, especially in the suborder *Melampsorineae* (Figure 3).

## Taxonomy

Family: *Chrysomyxaceae* Gäum. ex Leppik, Ann. Bot. Fenn. 9: 139. 1972

### Type genus

*Chrysomyxa* Unger, Beitr. Vergleich. Pathologie: 24. 1840.

### Type species

*Chrysomyxa abietis* (Wallr.) Unger, Beitr. Vergleich. Pathologie: 24. 1840. *Genus included in this family. Chrysomyxa* and *Rossmanomyces*. *Spermogonia* Group I (type 2), subepidermal, determinate, with flat hymenia, bounding structures lacking. *Aecia Peridermium*-type, subepidermal, with well-developed peridia, aeciospores catenulate, with intercalary cells, mainly verrucose. *Uredinia Caeoma*-type, subepidermal, erumpent, with or without inconspicuous peridia, wiitalicthout ostiolar cells, urediniospores with intercalary cells, germ pores scattered. *Telia* subepidermal, erumpent, teliospores aseptate, catenulate, crowded but loosely adherent, wall thin, germination occurs without dormancy. *Basidia* external or without basidiospores.

Notes – *Chrysomyxa* was previously placed in *Chrysomyxaceae, Coleosporiaceae*, or *Melampsoraceae* by different taxonomists based on various morphological criteria in teliospores (Sydow and Sydow 1915; Dietel 1928; Leppik 1972; Savile 1976). Cummins and Hiratsuka (1983) placed *Chrysomyxa* in *Coleosporiaceae* together with *Coleosporium* by emphasising the importance of spermogonial and teliospore’s morphologies, however, telial differences between the two genera have been clearly demonstrated (Sydow and Sydow 1915; Crane et al. 2005). *Chrysomyxa* was then placed in *Coleosporiaceae* by Aime and McTaggart (2021), along with *Coleosporium, Cronartium, Quasipucciniastrum, Rossmanomyces* and *Thekopsora*, but these genera in this broadly defined family have high morphological variations in the structures of spermogonia and telia, which had long been used as important criteria at the familial level (Cummins and Hiratsuka 2003).

The polyphyly of the family *Coleosporiaceae* defined by Aime and McTaggart (2021) has been revealed by our previous molecular phylogenetic analyses (Zhao et al. 2020, 2021). Our current findings further demonstrated the phylogenetic distinction of *Chrysomyxa* from *Coleosporium*, and two genera have different spermogonial, uredinial and telial morphologies (Figure 1). Furthermore, the divergence time of two genera and their median ages were in a general range of divergent time of families in *Basidiomycota* (Aime et al. 2018; He et al. 2019). All these findings supported the taxonomic treatments of Dietel and Neger (1900) and Leppik (1972), who placed the genus *Chrysomyxa* in the *Chrysomyxaceae*. The family name *Chrysomyxaceae* is therefore resurrected here. Aime and McTaggart (2021) established a new genus *Rossmanomyces*, which is phylogenetic allied to *Chrysomyxa* but differs in forming a systemic sporothallus in telia. Here we placed genera *Chrysomyxa* and *Rossmanomyces* in the family *Chrysomyxaceae*.

Family: *Coleosporiaceae* Dietel, in Engler and Prantl, Nat. Pflanzenfam., Teil. I (Leipzig) 1(1): 548. 1900, *emend*. P. Zhao and L. Cai

### Type genus

*Coleosporium* Lév., Annls Sci. Nat., Bot., Sér. 3 8: 373. 1847.

### Type species

*Coleosporium campanulae* (Pers.) Tul., Annls Sci. Nat., Bot., Sér. 42: 137. 1854.

*Genus included in this family. Coleosporium.*

*Spermogonia* Group I (type 2), subepidermal, determinate, with flat hymenia, bounding structures lacking. *Aecia Peridermium*-type, with well-developed peridia, aeciospores catenulate, with intercalary cells, verrucose. *Uredinia Caeoma*-type, with rudimentary peridia or none, without ostiolar cells, urediniospores echinulate, with intercalary cells, mostly verrucose. *Telia* erumpent as low or rarely columnar cushions, gelatinous, teliospores aseptate, sessile, catenulate, in 1-layered crusts or pseudocatenulate by intrusion of young spores among older ones. *Basidia* internal.

Notes – *Coleosporiaceae* has undergone multiple taxonomic reassignments over the years based on various morphological criteria in teliospores and spermogonia (Wilson and Henderson 1966; Hiratsuka et al. 1992; Cummins and Hiratsuka 2003). Recently, Aime and Taggart (2021) included seven genera in this family, despite the fact that the spore-producing structures (i.e. spermogonia, aecia, uredinia, telia and basidia) from those genera have broad morphological variations (Figure 1). Based on phylogenetic analyses and morphological comparisons with the spore-producing structures (Figure 1), we included two genera (i.e. *Chrysomyxa, Rossmanomyces*) in the newly resurrected family *Chrysomyxaceae*. The genus *Cronartium* was re-classified in the family *Cronartiaceae*, and genus *Thekopsora* was classified in the new family *Thekopsoraceae*. Thus, *Coleosporiaceae* is emended with a narrower concept based on the type genus *Coleosporium*, which is distinctive from other families in telia with internal basidia and unicellular teliospores in 1-layered crust, wall thick and gelatinising above (Figure 1).

Family: *Cronartiaceae* Dietel, in Engler and Prantl, Nat. Pflanzenfam., Teil. I (Leipzig) 1(1): 548. 1900

### Type genus

*Cronartium* Fr., Observ. Mycol. (Havniae) 1: 220. 1815.

### Type species

*Cronartium asclepiadeum* (Willd.) Fr., Observ. Mycol. (Havniae) 1: 220. 1815.

*Genera included in this family. Cronartium*.

*Spermogonia* Group II (type 9), intracortical, indeterminate, with flat hymenia, bounding structures lacking. *Aecia Peridermium*-type, the peridia large and blister-like, strongly developed, rupturing widely, aeciospores catenulate, with intercalary cells, verrucose with rod-like columns. *Uredinia Milesia*-type, subepidermal, opening by a pore, with ostiolar cells, urediniospores borne singly, echinulate, germ pore scattered. *Telia* with high variations, subepidermal, erumpent, vteliospores aseptate, crowded but loosely adherent, some catenulate, thin-walled, germination occurs without dormancy. *Basidia* external.

Notes – *Cronartium* was originally placed in the family *Pucciniaceae* (Dietel 1897), but then classified as a member of *Cronartiaceae, Coleosporiaceae, Melampsoraceae* or *Pucciniastraceae* in different time periods based on various morphological criteria in teliospores or spermogonia (Dietel 1928; Cummins and Hiratsuka 1983, 2003; Aime and Taggart 2021). Recent molecular phylogenetic studies confirmed the placement of *Cronartium* in the suborder *Melampsorineae* (Aime 2006; Aime et al. 2018), but its relationship with several other genera, including *Chrysomyxa, Coleosporium, Diaphanopellis, Quasipucciniastrum, Rossmanomyces* and *Thekopsora*, was still uncertain (Aime and Taggart 2021). Our current results and previous phylogenetic studies of the order *Pucciniales* (Zhao et al. 2020, 2021) clearly revealed the phylogenetic distinction between *Cronartium* and the above-mentioned genera (Figure 1), and *Cronartium* clade differs from other families in the spore-producing structures, especially in Group II spermonogia, *Milesia*-type uredinia with ostiolar cells, and telia with sessile, catenulate teliospores (Figure 1). Thus, here we resurrected the family *Cronartiaceae* to accommodate the genus *Cronartium.*

Family: *Hyalopsoraceae* P. Zhao and L. Cai, *fam. nov*. – MycoBank MB842413

### Etymology

Name derived from the type genus, *Hyalopsora.*

### Type genus

*Hyalopsora* Magnus, Ber. Dt. Bot. Ges. 19: 582. 1902.

### Type species

*Hyalopsora aspidiotus* (Peck) Magnus, Ber. Dt. Bot. Ges. 19: 582. 1901.

*Genus included in this family. Coleopuccinia, Hyalopsora, Melampsoridium.*

*Spermogonia* Group I (type 2 or type 3), subepidermal or subcuticular, determinate, with flat hymenia, bounding structures lacking. *Aecia Peridermium*-type, with peridium, subepidermal, aeciospore catenulate. *Uredinia Milesia*-type, subepidermal, with peridium opening by a discrete pore, with ostiolar cells, urediniospores borne singly, wall colourless, echinulate, germ pores scattered or bizonate. *Telia* intraepidermal, not erumpent, 2 to many-celled by septa, wall colourless, teliospores aseptate, sessile, pigmented. *Basidia* external.

Notes – The traditionally defined *Pucciniastraceae* has been revealed to be polyphyletic, with members dispersed across several distant and well-supported clades (Figure 1; Aime et al. 2018; Zhao et al. 2020). Three closely related clades, representing genera *Coleopuccinia, Hyalopsora* and *Melampsoridium*, show a high morphological consistency in the spore-producing structures with each other, and they can be distinguished from phylogenetically allied families in intraepidermal telia with sessile and unicellular teliospores with intercalary cell (Figure 1). Thus, a new family *Hyalopsoraceae* is proposed to accommodate *Coleopuccinia, Hyalopsora* and *Melampsoridium*. Previously, Cummins and Hiratsuka (2003) considered the genus *Coleopuccinia* as a synonym of *Gymnosporangium* (*Gymnosporangiaceae*) based on teliospores similarities, and Cao et al. (2018) recognised the phylogenetic distinctiveness of two genera. Here we confirm its familial position in *Hyalopsoraceae* for the first time, and our findings further highlight the importance of the spore-producing structures for familial delimitation.

Family: *Melampsoraceae* Dietel, in Engler and Prantl, Nat. Pflanzenfam., Teil. I (Leipzig) 1(1): 38. 1897

### Type genus

*Melampsora* Castagne (1843).

### Type species

*Melampsora euphorbiae* (Ficinus and C. Schub.) Castagne, Observ. Uréd. 2: 18.1843.

*Genus included in this family. Ceropsora* and *Melampsora.*

*Spermogonia* Group I (type 2 or type 3), subepidermal or subcuticular, determinate, with flat hymenia, bounding structures lacking. *Aecia Caeoma*-type, subepidermal, with rudimentary or no peridia, aeciospore catenulate, with intercalary cells. *Uredinia Uredo*-type, subepidermal, erumpent, without ostiolar cells, with abundant capitate paraphyses, urediniospores borne singly, echinulate, germ pores scattered or bizonate. *Telia* subepidermal or subcuticular, not erumpent, consisting of laterally adherent teliospores in crusts one spore deep or some species with subjacent spore-like cells, teliospores aseptate, sessile, pigmented. *Basidia* external.

Notes – Both *Ceropsora* and *Melampsora* are distinctive from the rest families in *Caeoma*-type aecia, *Uredo*-type uredinia, and telia with sessile and unicellular teliospores (Figure 1; Sydow and Sydow 1915; Cummins and Hiratsuka 2003; Aime and Taggart 2021). Two genera constitute the family *Melampsoraceae*, which is confirmed to be monophyly by Tian et al. (2004), Feau et al. (2009), and Zhao et al. (2017, 2020). This family was estimated to be diverged around 12 ~ 42 MYA (median ages 31.94 MYA), within the generally acknowledged divergent time range for families of *Basidiomycota* (Aime et al. 2018; He et al. 2019).

Family: *Milesinaceae* Aime and McTaggart, Fungal Systematics and Evolution 7: 32. 2020

*Genera included in this family. Milesina, Naohidemyces* and *Uredinopsis*.

*Spermogonia* Group I (type 1), subepidermal, with concave hymenia, bounding structures lacking. *Aecia Peridermium*-type, subepidermal, erumpent, with peridia, aeciospores catenulate, verrucose. *Uredinia Milesia*-type, subepidermal, urediniospores mostly echinulate, borne singly. *Telia* intraepidermal, consisting of spores in the epidermal cells, teliospores aseptate or multiseptate, with oblique septa. *Basidia* external.

Notes – *Milesina, Naohidemyces* and *Uredinopsis* were classified in *Pucciniastraceae* based on their teliospores that are embedded in the host tissue and the *Peridermium*-type aecia (Cummins and Hiratsuka 1984; Cummins and Hiratsuka 2003). Phylogenetically, *Milesia, Naohidemyces* and *Uredinopsis* clustered in one phylogenetic group, and separated from *Hyalopsora, Melampsorella, Melampsoridium, Pucciniastrum* and *Thekopsora* (Figure 1). In morphology, *Milesia, Naohidemyces* and *Uredinopsis* have similar morphological features in all spore-producing structures, but clearly differentiate themselves from other families in *Milesia*-type uredinia without ostiolar cells and wall colourless (Figure 1). Thus, Aime and McTaggart (2021) proposed *Milesinaceae* to accommodate these three genera. Our results is in agreement of Aime and McTaggart (2021).

Family: *Nothopucciniastraceae* P. Zhao and L. Cai, *fam. nov*. – MycoBank MB842414

### Etymology

*Notho *= nothus in Greek, fake, close but different; *pucciniastraceae *= *Pucciniastraceae*-like morphology.

### Type genus

*Nothopucciniastrum* P. Zhao and L. Cai, *gen. nov.*

### Type species

*Nothopucciniastrum tiliae* (Miyabe) P. Zhao and L. Cai, *comb. nov*.

*Genera included in this family. Nothopucciniastrum.*

*Spermogonia* Group I (type 2 and 3), subepidermal or subcuticular, determinate, with flat hymenia, bounding structures lacking. *Aecia Peridermium*-type, or *Milesia*-type, with well-developed peridia, aeciospores borne singly on pedicels, verrucose. *Uredinia Milesia*-type, with well-developed ostiolar cells, urediniospores borne singly, verrucose. *Telia* subepidermal, not erumpent, consisting of laterally adherent teliospores one spore deep, teliospores sessile, aseptate or multiseptate, with vertical septa. *Basidia* external.

**New combinations:**

Nothopucciniastrum actinidiae (Hirats. f.) P. Zhao and L. Cai, *comb. nov.*

*Basionym. Pucciniastrum actinidiae* Hirats. f., Mem. Tottori Agric. Coll. 4: 279. 1936.

Nothopucciniastrum boehmeriae ((Dietel) Syd. and P. Syd.) P. Zhao and L. Cai, *comb. nov.*

*Basionym. Pucciniastrum boehmeriae* (Dietel) Syd. and P. Syd., Ann. Mycol. 1(1): 19. 1903.

Nothopucciniastrum corni (Dietel) P. Zhao and L. Cai, *comb. nov.*

*Basionym. Pucciniastrum corni* Dietel, Bot. Jb. 34: 587. 1905.

Nothopucciniastrum fagi (Dietel) P. Zhao and L. Cai, *comb. nov.*

*Basionym. Pucciniastrum fagi* G. Yamada, Bot. Mag., Tokyo 44: 280. 1930.

Nothopucciniastrum kusanoi (Dietel) P. Zhao and L. Cai, *comb. nov.*

*Basionym. Pucciniastrum kusanoi* Dietel, Bot. Jb. 32: 629. 1903.

Nothopucciniastrum hikosanense (Hirats. f.) P. Zhao and L. Cai, *comb. nov.*

*Basionym. Pucciniastrum hikosanense* Hirats. f., Ann. Phytopath. Soc. Japan 10: 154. 1940.

Nothopucciniastrum miyabeanum (Hirats.) P. Zhao and L. Cai, *comb. nov.*

*Basionym. Pucciniastrum miyabeanum* Hirats., Bot. Mag., Tokyo 12: 3 (extr.). 1898.

Nothopucciniastrum styracinum (Hirats.) P. Zhao and L. Cai, *comb. nov.*

*Basionym. Pucciniastrum styracinum* Hirats., Bot. Mag., Tokyo 12: 2 (extr.). 1898.

Nothopucciniastrum tiliae (Miyabe) P. Zhao and L. Cai, *comb. nov.*

*Basionym. Pucciniastrum tiliae* Miyabe, in Hiratsuka, Bot. Mag., Tokyo 11: 47. 1897.

Nothopucciniastrum yoshinagae (Hirats.) P. Zhao and L. Cai, *comb. nov.*

*Basionym. Pucciniastrum yoshinagae* Hirats. f. [as “yoshinagai”], Trans. Tottori Soc. Agric. Sci. 2: 247. 1931.

Notes – The traditionally defined *Pucciniastraceae* with 9 genera (Cummins and Hiratsuka 1984; Cummins and Hiratsuka 2003) has been demonstrated to be highly polyphyletic (Figure 1; Aime et al. 2018; Zhao et al. 2021). Recent treatments of *Pucciniastraceae* have placed *Milesia, Naohidemyces* and *Uredinopsis* in *Milesinaceae* (Aime and McTaggart 2021), *Coleopuccinia, Hyalopsora*, and *Melampsoridium* in *Hyalopsoraceae*, and *Thekopsora* in *Thekopsoraceae* (this study). Furthermore, *Pucciniastrum* species were found to cluster in two distant clades (Figure 1). Ten species formerly classified into genus *Pucciniastrum*, (i.e. *P. actinidiae, P. boehmeriae, P. corni, P. fagi, P. hikosanense, P. kusanoi, P. miyabeanum, P. styracinum, P. tiliae* and *P. yoshinagai*) constituted one well-supported clade distinct from the clade of *Pucciniastrum* comprising the type species *P. epilobii* (Figure 1). This result is consistent with Qi et al. (2019), and Zhao et al. (2020). The morphological difference between these *Pucciniastrum* species in above two clades were discussed in Liang (2006) and Yang (2015), and those ten species different from *Pucciniastrum* and *Melampsorella* in their *Milesia*-type with ostiolar cells. Based on morphological and molecular evidences, we propose a new family *Nothopucciniastraceae* and a new genus *Nothopucciniastrum* to accommodate these ten species which are herein treated as new combinations. Results further emphasised the importance of uredinial morphologies for familial delimitation.

Family: *Pucciniastraceae* Gäum. Ex Leppik, Ann. Bot. Fenn. 9: 139. 1972, *emend*. P. Zhao and L. Cai

### Type genus

*Pucciniastrum* G.H. Otth (1861).

### Type species

*Pucciniastrum epilobii* (Pers.) G.H. Otth, Mitt. Naturf. Ges. Bern 469–496: 72. 1861.

*Genera included in this family. Melampsorella* and *Pucciniastrum.*

*Spermogonia* Group I (type 2 and 3), subepidermal or subcuticular, determinate, with flat hymenia, bounding structures lacking. *Aecia Peridermium*-type, or *Milesia*-type, with well-developed peridia, aeciospores borne singly on pedicels, verrucose. *Uredinia Milesia*-type, without ostiolar cells, urediniospores borne singly, verrucose. *Telia* subepidermal or intraepidermal, not erumpent, consisting of laterally adherent teliospores one spore deep, teliospores sessile, aseptate or ultiseptated, with vertical septa. *Basidia* external.

Notes – With the segregation of *Coleopuccinia, Hyalopsora, Melampsoridium silesia, Naohidemyces, Quasipucciniastrum, Thekopsora, Uredinopsis* from *Pucciniastraceae*, we herein redefine the *Pucciniastraceae* with a narrower concept which include genera *Melampsorella* and *Pucciniastrum. Pucciniastraceae* is distinctive from the rest families by the absence of ostiolar cells in *Milesia*-type uredinia (Figure 1).

Family: *Thekopsoraceae* P. Zhao and L. Cai, *fam. nov*. – MycoBank MB842415

### Etymology

Name derived from the type genus, *Thekopsora.*

### Type genus

*Thekopsora* Magnus, Hedwigia 14: 123. 1875.

### Type species

*Thekopsora areolata* (Fr.) Magnus, Sber. Gesellschaft Naturf. Freunde Berlin: 58. 1875.

*Genera included in this family. Thekopsora.*

*Spermogonia* Group I (type 2 and 3), subepidermal or subcuticular, determinate, with flat hymenia, bounding structures lacking. *Aecia Peridermium*-type, or *Milesia*-type, with well-developed peridia, aeciospores borne singly on pedicels, verrucose. *Uredinia Milesia*-type, with well-developed ostiolar cells with apparent spines, urediniospores borne singly, verrucose. *Telia* intraepidermal, not erumpent, consisting of laterally adherent teliospores one spore deep, teliospores sessile, aseptate or multiseptated, with vertical septa. *Basidia* external.

Notes – The *Thekopsora* clade, including the type of the genus, *Thekopsora areolata*, was phylogenetically close to *Cronartium* but distinct from *Pucciniastrum* species (Figure 1), in agreement with Aime et al. (2018). In morphology, it resembles *Coleopuccinia, Hylospora, Melampsoridium*, and *Pucciniastrum*, but differs from these genera in the aecia, uredinia and telia (Figure 1; Yang 2015). It also differs from the phylogenetically allied family *Cronartiaceae* in the structures of spermogonia, uredinia and telia. Thus, a new family *Thekopsoraceae* is proposed to accommodate the genus *Thekopsora.*

## Discussion

### Phylogenetic reappraisal of rust families and genera

To date, more than 7 800 rust species have been described in 289 genera in *Pucciniales* (Laundon 1965; Kirk et al. 2008). Cummins and Hiratsuka (2003) in their monograph “Illustrated Genera of Rust Fungi” included 120 holomorphic genera and 13 asexual typified genera, in which most genera are traditionally morphologically defined. At the family level, since the first system proposed by Dietel (1897), different mycologists have classified 289 recognised genera into 2–14 families mainly based on morphological characters in spermogonia and teliospores (Arthur 1907, 1934; Kuprevich and Tranzschel 1957; Wilson and Henderson 1966; Azbukina 1972; Cummins and Hiratsuka 1984; Buriticá 1991; Hiratsuka et al. 1992; Cummins and Hiratsuka 2003). Although urediniologists have generally accepted Cummins and Hiratsuka (2003)’s taxonomy system, subsequent studies have found numerous inconsistencies between this system and the molecular phylogeny (Wingfield et al. 2004; Aime 2006; Aime et al. 2018; Zhao et al. 2020). A definite family level resolution has not been obtained due to the lack of a comprehensive molecular phylogenetic study within the order *Pucciniales*. In our previous studies on the whole order, we proposed four new families based on extensive phylogenetic and morphological comparisons, and also recognised inconsistencies between morphologically-defined families in *Melampsorineae* suborder and their molecular phylogenetic relationships (Zhao et al. 2020, 2021). Thus, we conducted phylogenetic studies of 16 genera in the *Melampsorineae* suborder, and confirmed boundaries at the familial and generic level. Traditional morphology-defined families such as *Coleosporiaceae, Chrysomyxaceae*, and *Melampsoraceae* have been shown to be monophyletic with the core of species around the type (Figure 1). *Pucciniastraceae* has been found to be polyphyletic and its traditional members scattered in numerous discrete lineages. This family has been redefined by emphasising the importance of traditional taxonomic criteria used by Cummins and Hiratsuka (2003) and Aime and McTaggart (2021). In this study, we further emphasised several newly recognised criteria for familial delineation, i.e. the structures of uredinia and telia, including the colour of urediniospores, the existence of peridia in uredinia and telia, the existence of ostiolar cells in uredinia and their ornamentations. Our phylogenetic studies further emphasised that morphologies throughout their life cycles, especially the uredinial and aecial morphologies, are of great diagnostic value in delineating at the familial and generic level.

### Importance of applying early diverged characters in higher rank taxonomy

Hitherto, classification of the rust fungi at species, generic and family levels relies on morphological features of different spores and spore-producing structures in different stages throughout the life cycles. Spore morphologies, such as basidiospores, spermatia, aeciospores, urediniospores and teliospores, as well as spore-producing structures like basidia, spermogonia, aecia, uredinia and telia, were hitherto not seriously investigated to see if they are phylogenetically significant at particular taxonomic levels.

Our previous and current studies on the phylogeny of *Pucciniales*, particularly those in the *Melampsorineae*, have revealed that morphological characters, especially spore-producing structures throughout the whole life cycle, such as basidia spermogonia, aecia, uredinia and telia, were phylogenetically more informative at higher taxonomic ranks (family level). By contrast, spore morphologies such as basidiospores, spermatia, aeciospores, urediniospores and teliospores, were phylogenetically more informative at lower taxonomic rank (species level) (Tian et al. 2004; Crane et al. 2005; Feau et al. 2009, Beenken 2014, 2017; Zhao et al. 2015, 2017). These spore-producing structures represent early diverged morphological characters, while spore morphologies have already been proved to be recently diverged characters by our continuous investigations (Figure 1; Zhao et al. 2020, 2021). Our findings were further supported by the successive evolutionary process of rust fungi, because the structures of basidia, spermogonia, aecia, uredinia and telia might be directly influenced at early adaptation stages of rust fungi when they shifted from ferns to conifers and angiosperms (Leppik 1965). Based on these findings, taxonomic significance of morphological features in different spore stages throughout the whole life cycle in the rust fungi is proposed as shown in Figure 4. It is quite obvious that the polyphylies of many traditionally defined genera and families were resulted from the inappropriate use of recently diverged characters (particularly morphology of teliospores) at higher level taxonomy, and the use of the early diverged characters at lower-level taxonomy. Apparently these recently diverged characters have evolved more than once in different lineages during the evolutionary process.

Among the early diverged characters, the structures of spermogonia and telia have long been employed for classification at the family level, but the structures of aecia and uredinia have long been overlooked at higher taxonomic ranks (Hiratsuka and Cummins 1963; Hiratsuka and Hiratsuka 1980; Cummins and Hiratsuka 1983, 2003). Our studies indicated the importance of aecia and uredinia morphology for higher level classification, especially differences in spore ontogeny, hymenium shape, position in host tissues, presence of intercalary cells and paraphyses. Until now, fourteen different morphological types in aecial and uredinial structure have been recognised (Kenny 1970; Sato and Sato 1985). These morphological variations appear to be very useful criteria at family and generic level taxonomy in rust fungi.
